# Dendrochronological dates confirm a Late Prehistoric population decline in the American Southwest derived from radiocarbon dates

**DOI:** 10.1098/rstb.2019.0718

**Published:** 2020-11-30

**Authors:** Erick Robinson, R. Kyle Bocinsky, Darcy Bird, Jacob Freeman, Robert L. Kelly

**Affiliations:** 1 Department of Sociology, Social Work, and Anthropology, Utah State University, 0730 Old Main Hill, Logan, UT 84322-0730, USA; 2 Crow Canyon Archaeological Center, 23390 Road K, Cortez, CO 81321, USA; 3 Department of Anthropology, Washington State University, College Hall 150, PO Box 644910, Pullman, WA 99164-4910, USA; 4 Department of Anthropology, University of Wyoming, 1000 E. University Ave, Laramie, WY 82070, USA

**Keywords:** dates-as-data, radiocarbon summed probability distributions, dendrochronology, palaeodemography, Southwest USA

## Abstract

The northern American Southwest provides one of the most well-documented cases of human population growth and decline in the world. The geographic extent of this decline in North America is unknown owing to the lack of high-resolution palaeodemographic data from regions across and beyond the greater Southwest, where archaeological radiocarbon data are often the only available proxy for investigating these palaeodemographic processes. Radiocarbon time series across and beyond the greater Southwest suggest widespread population collapses from AD 1300 to 1600. However, radiocarbon data have potential biases caused by variable radiocarbon sample preservation, sample collection and the nonlinearity of the radiocarbon calibration curve. In order to be confident in the wider trends seen in radiocarbon time series across and beyond the greater Southwest, here we focus on regions that have multiple palaeodemographic proxies and compare those proxies to radiocarbon time series. We develop a new method for time series analysis and comparison between dendrochronological data and radiocarbon data. Results confirm a multiple proxy decline in human populations across the Upland US Southwest, Central Mesa Verde and Northern Rio Grande from AD 1300 to 1600. These results lend confidence to single proxy radiocarbon-based reconstructions of palaeodemography outside the Southwest that suggest post-AD 1300 population declines in many parts of North America.

This article is part of the theme issue ‘Cross-disciplinary approaches to prehistoric demography’.

## 1. Introduction

The spatial and temporal ubiquity of archaeological radiocarbon dates makes them an unprecedented proxy for human palaeodemography. The past decade has witnessed a rapid increase in the use of archaeological radiocarbon time series to reconstruct prehistoric population growth and decline in various regions of the world [1–9]. One of the first aggregations of radiocarbon dates for palaeodemographic analysis was by Berry [10] in his study of population growth, decline and migration across the American Southwest. Radiocarbon time series were used as a complement to earlier periods that lacked robust dendrochronological records. Berry [10, p. 120] noted that they were ‘not even remotely related to population size’, but rather the ‘*relative probability* of occupation through time’ and ‘the major trends in these charts (i.e. peaks and troughs) should provide a reliable indication of the *direction* of change’. Later, Rick [11, p. 56] more formally outlined the use of radiocarbon time series as a method for palaeodemography by developing an ‘inference chain’ that detailed the various biases involved in their use and interpretation. Rick identified three biases [11]: creation bias, preservation bias and investigation bias. Over the past decade, as archaeologists have assembled and analysed large radiocarbon datasets from various regions of the world, there has been considerable focus on these biases, notably creation [12–14] and preservation bias [5,9,15–17], as well as bias caused by the radiocarbon calibration process [9,18–22]. Of the three biases Rick identified, investigation bias has been the most understudied thus far. Investigation bias occurs when investigators gather more radiocarbon samples for particular periods of time or specific geographic regions, which leads to periods or regions with more or less radiocarbon representation than the average time period or region. The biased selection of radiocarbon samples would then appear to be relative increases or decreases in population when they are merely artefacts of research history and fieldwork practice. Investigation bias has been understudied because for many regions of the world radiocarbon data are the only currently available proxy evidence enabling reconstructions of palaeodemography in continuous time series spanning millennia. Assessing the impact of investigation bias on radiocarbon-based reconstructions of palaeodemography requires comparison with other palaeodemographic proxies.

The American Southwest provides one of the best available case studies for assessing the role of investigation bias in radiocarbon-based reconstructions of palaeodemography. It also has one of the most well-documented cases of population growth and decline in the world [23–27]. For almost a century, researchers have collected multiple proxies, such as dendrochronological records, settlement size records and ceramic seriation records to address the magnitude of this population growth and decline. However, a critical knowledge gap still exists for the exact scale of population decline across and beyond the Southwest [28,29]. This gap is because the less intensively researched regions lack multiple proxies for robust reconstructions of palaeodemography. In these regions, radiocarbon data are the only available proxy time series for reconstructing palaeodemography. Over the past 6 years, we have collected all available radiocarbon data across and beyond the Southwest [26]. Initial analyses of this new radiocarbon dataset have revealed extensive population decline from AD 1300 to 1600 across and beyond the Southwest [26,27]. Therefore, the geographic extent of Late Prehistoric population decline could be much broader than previously expected.

The potential importance of this widespread population decline for research on Late Prehistoric archaeology of the American West requires the comparison of the sensitivity of lower resolution radiocarbon data against other higher resolution palaeodemographic proxies. In the most well-documented regions (such as the northern American Southwest), we expect radiocarbon records to reflect the highest amount of investigation bias, as researchers have the option of selecting from a suite of other higher resolution proxies, including dendrochronology and ceramic seriation. Investigation bias can create an artificial ‘edge effect’ spuriously appearing as a decrease in population. Here, we develop a new method for arraying dendrochronological records as time series for comparison against radiocarbon time series. We focus on the broader region of the Upland US Southwest (UUSW) for which there is a robust dendrochronological record [23].

**Figure 1.**
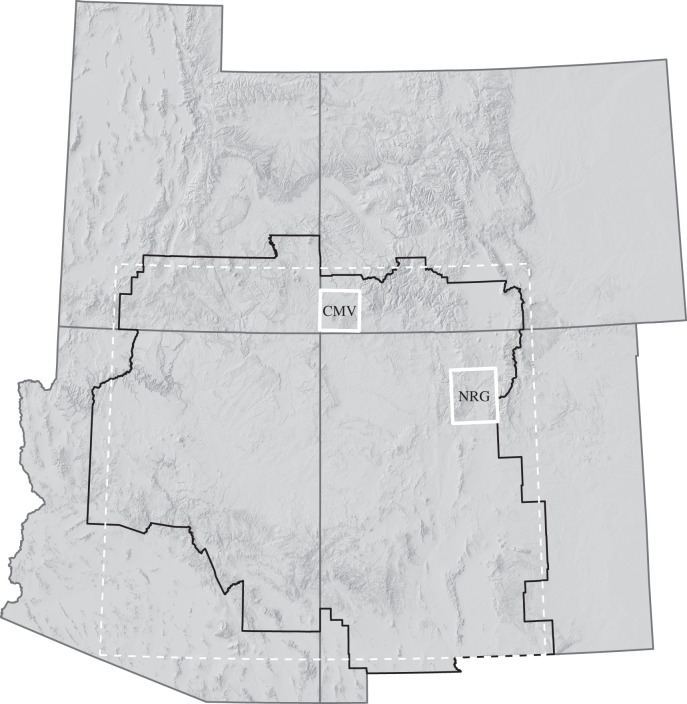
The UUSW as defined in this study (region with black border). The white dashed area represents the UUSW as defined in [23]. VEP study areas are white boxes. CMV: Central Mesa Verde; NRG: Northern Rio Grande.

We also focus on the more concentrated and intensively investigated Central Mesa Verde (CMV) and Northern Rio Grande (NRG) regions of the UUSW (figure 1), for which multi-proxy palaeodemographic reconstructions were produced by the Village Ecodynamics Project (VEP) [30,31]. The VEP is an interdisciplinary, multi-method research initiative aimed at better understanding demography of ancestral Pueblo farmers and human–environment interaction across the UUSW. As part of the project, VEP researchers catalogued data on all known archaeological habitation sites within a 4600 km^2^ area in the CMV [31] and a 6900 km^2^ area in the NRG [30], to which most of the CMV population migrated during the thirteenth century AD [30]. They integrated data including pit structure and room count estimates, the presence of diagnostic architectural features, surface ceramic tallies, surveyor assessments and limited dendrochronological data in an empirical Bayesian framework to develop population estimates for each region resolved to periods ranging from 20 to 125 years in length [30,31]. The VEP estimates are the highest quality regional palaeodemographic reconstructions currently available in the UUSW and are completely independent of the radiocarbon records for the region. They are therefore useful in exploring the impact of investigation bias in the radiocarbon record.

We expect that substantial investigation bias is present in radiocarbon records if those records substantially diverge from the dendrochronological and multi-proxy reconstructions. If the lower resolution radiocarbon records reveal similar trends to the two other records, we expect investigation bias does not substantially impact the ability of radiocarbon records to be used as relative measures of the directionality of population change through time. This would, in turn, lend more confidence to radiocarbon records across the American West that reveal extensive post-AD 1300 population declines but lack other palaeodemographic proxies.

## 2. Methods

Here, we compare two large collections of radiocarbon and tree-ring dates drawn from the UUSW. After harmonizing those datasets with one another and calibrating the radiocarbon dates, we calculate binned summed probability distributions across the UUSW, CMV and NRG. The radiocarbon dates we analyse here, which have been uploaded to the Canadian Archaeological Radiocarbon Database (CARD; https://www.canadianarchaeology.ca), are drawn from the National Science Foundation supported project, ‘Populating a Radiocarbon Database for North America’. Tree-ring data are those presented in [23]. Both datasets are available on the Digital Archaeological Record (see Data accessibility, below).

### (a) Study area

The UUSW was geographically defined by [23] as the region between 105–113°W and 32–38°N. Here, we refine that definition in two ways. First, because location data for our radiocarbon database are only resolved to the county level for most sites [26], we define our study region to include all counties whose geographic centroids fall within the region from [23]. Second, because of the generally poor tree-ring date record in the Sonoran Desert, we exclude the Phoenix and Tucson basins by dropping Maricopa, Pinal and Pima counties in Arizona. Figure 1 shows the original UUSW from [23] and revised study area used in this analysis, as well as the two VEP study areas.

### (b) Period of interest

Here, we focus our analyses on the period for which data are most abundant: AD 200–1800. This period includes the periods of the VEP population estimates for the CMV (AD 600–1280) and NRG (AD 900–1760) and extends 400 years earlier than the CMV estimates to allow us to assess trends in the radiocarbon record leading up to the VEP reconstructions, and to control for edge effects. While the VEP demographic estimates are for ancestral Pueblo populations only, it is likely that the tree-ring records include samples from Spanish colonial contexts after AD 1600 or so.

### (c) Datasets

We cleaned the radiocarbon database by first removing all non-archaeological dates. To ensure our data are reasonably precise, we removed all radiocarbon dates with 1*σ* errors greater than 300 years and more than 25% of the uncalibrated age. We removed dates with duplicate laboratory numbers whose radiocarbon ages, 1*σ* errors, or county information did not match. We then trimmed these data to counties within the UUSW. We retained radiocarbon dates that likely derive from our period of interest by first calibrating all of the dates, then retaining dates whose 95% calibrated probability masses overlapped 1750–150 BP.

We draw on the tree-ring date database presented in [23]. These data were compiled by Tim Kohler and Rebecca Higgins from many contributors, and the vast majority of dates were produced by the Laboratory of Tree Ring Research at the University of Arizona. The original dataset contained 32 863 cutting, near-cutting and non-cutting dates from across the US Southwest. Here, we only use the dates that are most likely to be accurate—cutting dates (‘B’, ‘G’, ‘L’, ‘c’, or ‘r’ dates, with or without a ‘+’) for which we know the year the tree died, and near-cutting dates (‘v’ or ‘v+’ dates) that are likely within 0–3 years of the true date of the outermost ring. The [23] data contain site locations; for this analysis, we first spatially cropped the database to our study area, and then translated those locations to the county level. We converted the original date determinations to BP to match calibrated radiocarbon year conventions and retained dates within 1750–150 BP.

Finally, owing to much of the radiocarbon data only being resolved to the county level, we had to approximate inclusion in the VEP, CMV and NRG study areas. The CMV study area conforms well to Montezuma county, Colorado; the NRG overlaps with Los Alamos, Rio Arriba, Sandoval, Santa Fe and Taos counties in New Mexico. In the analyses below, dates were included as ‘within’ each study area if they derive from those respective counties.

Our final dataset includes 1531 radiocarbon dates from 482 unique sites, dating from 2260 to 90 BP (uncalibrated; 309 BC–AD 1860) and 15 077 tree-ring dates from 770 unique sites, dating from 1749 to 152 BP (201–1798 AD). Table 1 presents the counts of dates and sites of each date type in the CMV and NRG study areas.

**Table 1.** Counts of radiocarbon and tree-ring dates and sites in this study.

study area	radiocarbon		tree-ring	
	dates	sites	dates	sites
UUSW	1531	482	15 077	770
CMV	36	18	4435	219
NRG	83	40	2306	146

**Figure 2.**
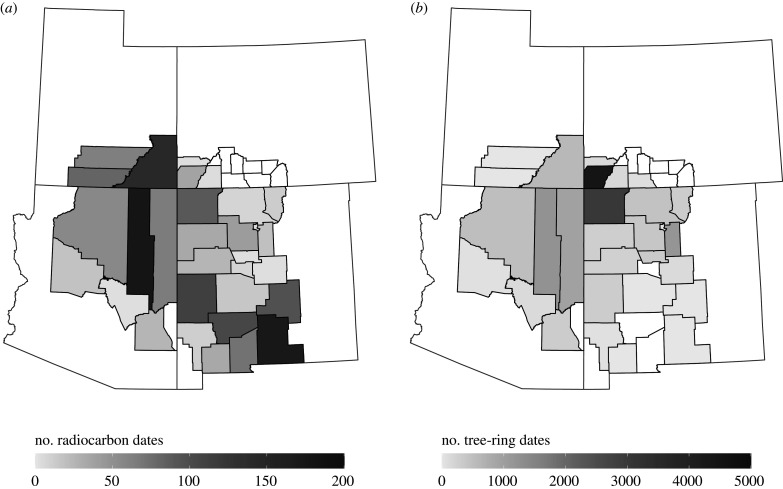
The number of radiocarbon and tree-ring dates by county in this study.

**Figure 3.**
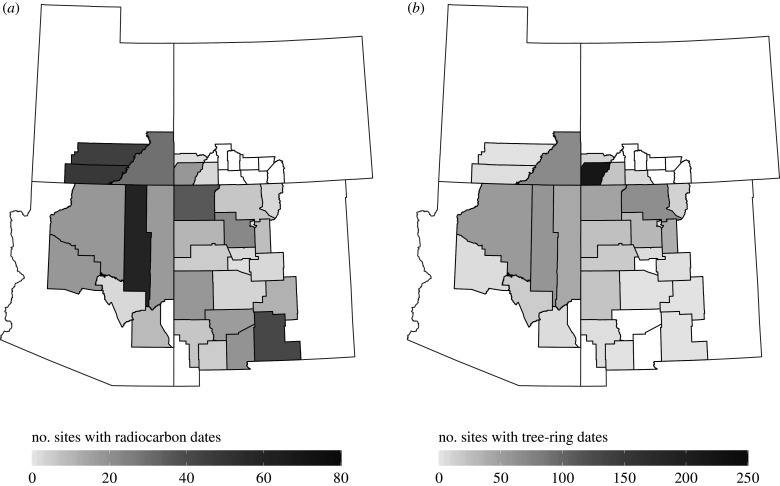
The number of sites with radiocarbon and tree-ring dates by county in this study.

Date densities across the study area were very uneven, with high counts of radiocarbon dates deriving from Navajo County, Arizona, and almost a third of the tree-ring dates in our study being from Montezuma County, Colorado. Below, we overcome some of these sampling issues by binning dates by site and occupation phase. Still, these disparities likely demonstrate more fundamental differences in research design across archaeological projects. There are very few radiocarbon dates from the two VEP study areas, indicating a clear bias towards using tree-ring dating where (and when) possible. Figure 2 presents the number of radiocarbon and tree-ring dates by county within our study area. Figure 3 presents the number of unique sites with radiocarbon and tree-ring dates by county within our study area. There are similar ratios of radiocarbon dates to sites with radiocarbon dates for the CMV and NRG (roughly 2 : 1), but sites in the CMV tend to have a third more tree-ring dates than those in the NRG (20 : 1 versus 15 : 1, respectively).

### (d) Preparing the summed probability distributions

To prepare the radiocarbon dataset, we used functions provided by the *rcarbon* package for *R* [32]. We calibrated the radiocarbon dates using the methods of [33], using the ‘IntCal13’ calibration curve [34]. To control for the inconsistent number of dates among the archaeological sites represented in the database (a form of investigation bias), we aggregated radiocarbon dates known to be from the same phase of an archaeological site using the *rcarbon::binPrep* function [32] using the single linkage agglomeration method with an h-value of 100 (see electronic supplementary material for a discussion of various h-values). This creates clusters of dates from the same site whose mean values (uncalibrated) are within 100 years of each other, and splits site occupations into distinct phases separated by 100 years or more. We constructed summed probability distributions (SPDs) from the calibrated dates using the *rcarbon::spd* function [32]. We averaged probability distributions of binned dates and then constructed SPDs from the resulting site-phase probability distributions.^1^

We treated the tree-ring dates in as similar a way possible to the radiocarbon dates. We modelled faux-calibrated tree-ring date probabilities as discrete degenerate distributions (*Pr*(*X* = *k*_0_) = 1), with *k*_0_ equal to the tree-ring date—in other words, a probability of 1 at the date value and 0 elsewhere. This is akin to modelling the tree-ring date as a normal distribution with a mean of the date value and a standard deviation of zero. Thinking of tree-ring dates this way allows us to use the same binning and SPD algorithms as for radiocarbon dates.^2^ Here, we binned the tree-ring dates into site-phases using the *rcarbon::binPrep* function and constructing SPDs using *rcarbon::spd*, both with the same parameter selections as used for the radiocarbon samples. As above, we averaged probability distributions of binned dates and then constructed SPDs from the site-phase distributions.

## 3. Results

**Figure 4.**
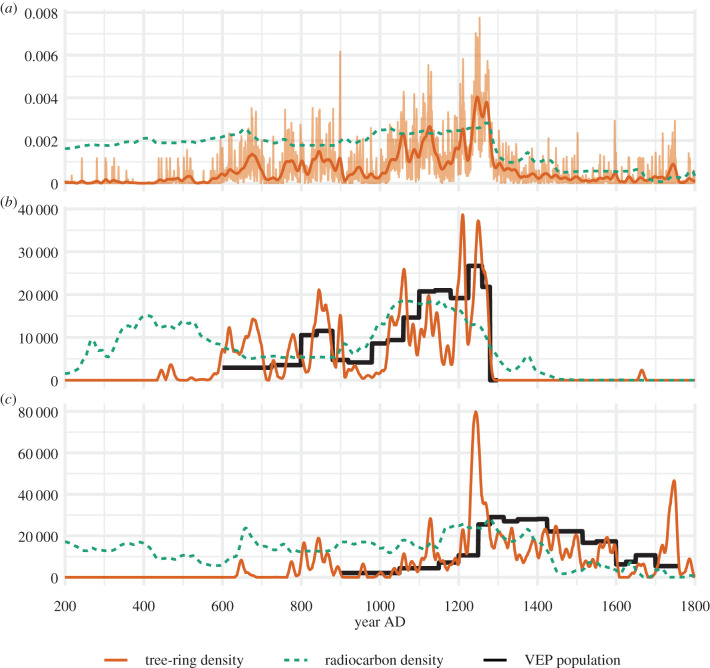
Radiocarbon and tree-ring density distributions in the UUSW (*a*), CMV (*b*), and NRG (*c*) regions, and VEP population estimates for the CMV and NRG. The dark orange tree-ring density curve smooths the raw tree-ring density (light orange) using a 21-year centre-aligned Gaussian kernel with a 5-year standard deviation, following [23]. In (*a*), the radiocarbon density (*y*-axis) is scaled by a factor of 3 to facilitate comparison with the tree-ring density over the target time period. The radiocarbon and tree-ring densities (*y*-axes) in (*b*) and (*c*) are scaled such that the area under their densities is equal to the area under the VEP population estimates (i.e. the cumulative population). (Online version in colour.)

Figure 4 presents radiocarbon and tree-ring SPDs for the UUSW, CMV and NRG regions, as well as VEP population reconstructions for the CMV and NRG regions. Across the UUSW, we see striking agreement in directionality and timing between the radiocarbon and tree-ring series (figure 4*a*); both series trend upwards until the late thirteenth century, and then decline rapidly. The tree-ring series—the light orange line is the raw SPD, and the dark line is smoothed—shows more variation on annual to decadal timescales and yet matches the broad trends of the radiocarbon SPD. Roughly 150-year periodic fluctuations in the tree-ring SPD, described as periods of ‘exploration’ and ‘exploitation’ by [23], are not present in the radiocarbon SPD, the result of a much smaller sample size of site-phases and lower temporal resolution of radiocarbon dating relative to tree-ring dating.

Comparisons between the CMV and NRG regional SPDs and the VEP population estimates are also revealing. In figure 4*b* and *c*, the regional SPDs have been scaled such that the areas under their densities (the orange and green-dotted lines) are equal to the area under the VEP population estimates (the black lines). For both regions, the radiocarbon and tree-ring SPDs capture the broad directionality of the VEP population estimates. In the CMV region (figure 4*b*), the radiocarbon SPD has substantial probability mass prior to the VEP reconstruction, which is likely owing to sampling bias in the radiocarbon record that is skewed in this region towards older (pre-Pueblo) sites. The radiocarbon SPD also does not capture the Pueblo I (AD 750–900) population peak (which may be too short-lived to be captured by such a small sample of radiocarbon dates) and seems to begin declining by the early thirteenth century AD. The tree-ring SPDs better capture the variability in the population, though include a decline in the mid AD 1100s that is not in the VEP population estimates. In the NRG region (figure 4*c*), both SPDs rise until about AD 1250, then decline thereafter. The high peak in the tree-ring record at AD 1250, far in excess of the VEP population estimate, is particularly interesting—it likely signals the net impact of the immigration of many thousands of people to the NRG region, and the resulting (and simultaneous) burst in construction. Such a sudden and synchronous demographic and architectural event is hard to capture in the relatively lower resolution and sparsely sampled radiocarbon record, which tends to be smeared across sub-centennial scales courtesy of the calibration process [35].

These results yield key insights on the relative sensitivity of the radiocarbon record to capture long-term, coarse-grained demographic change across the Southwest. As stated in §1, our aim is to understand how well the radiocarbon record captures population declines across the Southwest, as we need to develop a measure of confidence in radiocarbon records from regions across and beyond the Southwest that lack other demographic proxies. Both the radiocarbon and tree-ring SPDs show declines post-AD 1300 across all regions. In both the CMV and NRG, the decline in the radiocarbon SPD begins earlier than in the VEP population series. This may indicate that radiocarbon time series make the decline seem to begin earlier than it actually does, which could be caused by a ‘cliff’ in the radiocarbon calibration curve from AD 1200 to 1300 that is surrounded by two plateaus at AD 1100–1200 and AD 1400 [34].

Comparisons of the radiocarbon and dendrochronological record with the architecture/ceramic record show similarities and differences that reinforce the need for multiple proxy studies of palaeodemography when moving between broader and finer spatial scales. While the multi-proxy population reconstruction for the CMV region was similar to the radiocarbon and dendrochronological records, it showed a different trend for the NRG region (figure 4*c*). There, the population reconstruction peaks from AD 1300 to 1400, whereas the radiocarbon record declines, at first quickly and then slowly. These different trends between the VEP population reconstructions and dendrochronological and radiocarbon proxies reflect some investigation bias in both the radiocarbon and dendrochronological records at smaller, intra-regional scales, which calls for caution in the use of radiocarbon records to assess possible palaeo-migration scenarios at finer spatial scales. This result reinforces other work that has called for the use of multiple proxies as cross-checks on radiocarbon time series [9,12].

The central aim in comparing radiocarbon to dendrochronological records here was to assess the potential impacts of investigation bias in radiocarbon records, caused by researchers not taking radiocarbon samples in favour of other types of chronometric techniques (e.g. diagnostic artefact counts, tree-ring dates). Specifically, we are interested in the potential impact of this kind of bias in the post-AD 1300 decline of radiocarbon SPDs across and beyond the Southwest. Similarities in the decline of both radiocarbon and dendrochronological records across the UUSW suggest that, at this particular regional scale, radiocarbon records reflect long-term, coarse-grained trends in palaeodemography. Indeed, not only do we see corresponding declines in the radiocarbon and dendrochronological records at this large scale, but these patterns are also consistent with previous work on demographic and social change in the American Southwest: the Coalescent Communities Database, a Southwest-wide database of room counts and occupation spans of habitation sites with 12 rooms or more, supports a ‘population decline that began between AD 1300 and 1350 and continued to European contact’ (Hill *et al*. [24], figs 2 and 4) [36].

## 4. Conclusion

Understanding the geographic scale of post-AD 1300 human population declines in the American Southwest requires a reliance on radiocarbon-based reconstructions of palaeodemography, as radiocarbon is often the only proxy record available in many regions for developing continuous time series. However, radiocarbon records may include investigation bias caused by researchers not selecting radiocarbon samples in favour of Late Prehistoric diagnostic artefacts or other dating methods. In this paper, we develop a new method for systematically comparing radiocarbon time series against multiple palaeodemographic proxies. We selected three regions—the Upland US Southwest, Central Mesa Verde, and the Northern Rio Grande—where we expected the most investigation bias to occur, because in these regions multiple higher resolution dating techniques are available. Results indicated that all three palaeodemographic proxies reveal post-AD 1300 population declines. While there is some variability at finer spatial and temporal scales that warrants the collection of other data that can be used to cross-check and validate the resolution of radiocarbon records, at coarse-grained, large spatial and temporal scales radiocarbon records provide a robust relative measure of long-term palaeodemographic trends. These results lend confidence to single proxy radiocarbon-based reconstructions of palaeodemography outside the Southwest that suggest post-AD 1300 population declines in many parts of North America.

Investigation bias is an inevitable issue that all researchers using radiocarbon records have to address. The results of this paper advance our confidence in radiocarbon records as a coarse-grained measure of the directionality of human palaeodemographic trends. The methods developed can be used for other regions of the world that have dendrochronological records, such as Europe [37–39], where comparisons with radiocarbon records could help to advance knowledge of Neolithic ‘booms and busts' of populations [7,40] and to overcome limitations of the Bronze Age radiocarbon calibration plateau [18].

## Supplementary Material

Supplementary material

## Endnotes

1 Using this method, a site-phase with a single date and a site-phase with 10 dates would each contribute equally to the resulting SPD. This method of developing the SPD is therefore a model of the relative abundance of site occupation events through time rather than of dates through time. This method does not account for investigation biases deriving from the definition of ‘site’ or the decision to use a particular dating technique at a site in the first place.

2 One might consider alternative probability distributions for non-cutting or near-cutting dates. The probability mass function in such a case would be zero for years prior to the date of the outer ring of the sample and non-zero for subsequent years, presumably decaying quickly. For instance, we could imagine a study that derives empirical probability distributions for non-cutting dates based on cutting-dates from the same contexts.

## Data accessibility

A compendium of data and code used in this analysis is available on Github at https://github.com/people3k/robinson2020 and is archived with Zenodo at https://doi.org/10.5281/zenodo.3647599. Radiocarbon data used in this analysis are archived with the Digital Archaeological Record (tDAR) and are available at https://doi.org/10.6067/XCV8455460. Tree-ring data used here are archived with tDAR at https://doi.org/10.6067/XCV82J6D7B. Archaeological site locations are protected by US federal and state statutes. In order to protect these sensitive data, we pre-processed both the radiocarbon and tree-ring data; data included in the Github repository do not include site locations, and researchers will need to gain permission from the State Historic Preservation Office (SHPO) of each state in order to access the site location data.
