# Supplementary material for: Dendrochronological dates confirm a Late Prehistoric population decline in the American Southwest derived from radiocarbon dates
Source: Philos Trans R Soc Lond B Biol Sci. 2020 Nov 30;376(1816):20190718. doi: 10.1098/rstb.2019.0718 (PMC7741101; doi:10.1098/rstb.2019.0718)
Supplement: Supplementary material [file rstb20190718supp1.docx]

Supplementary Material for:

Dendrochronological dates confirm a Late Prehistoric

population decline in the American Southwest derived from

radiocarbon dates

Erick Robinson^1,*^, Kyle Bocinsky^2,3^, Darcy Bird^4^, Jacob Freeman^1^, and Robert L. Kelly^5^

^1^ Utah State University
^2^ Crow Canyon Archaeological Center
^3^ University of Montana
^4^ Washington State University
^5^ University of Wyoming

^*^ Correspondence: [Erick Robinson <erick.robinson@usu.edu>](mailto:erick.robinson@usu.edu)

# Cut-width sensitivity for temporal binning

Uneven sampling across archaeological contexts is a persistent problem when constructing summed probability distributions from radiocarbon dates, and perhaps even a larger issue for the more ubiquitous tree-ring dates in regions like the Upland US Southwest. In SPDs of radiocarbon dates, it is conventional to combine dates from single sites prior to creating the SPDs. Dates from separate components or phases of multi-component sites should be counted separately, however.

To control for inconsistent sample size across the archaeological sites represented in the database, we aggregated dates known to be from the same phase of same archaeological site using the rcarbon::binPrep function using the “single linkage” agglomeration method with a cut width (or $h$-value) of 100 [1]. This clusters dates from the same site that are within 100 years of each other, and splits site occupations into distinct phases separated by 100 years or more. Binned calibrated dates are then summed, and the resulting probability mass function is divided by the number of dates in the site-phase.

Because the wider archaeological SPD research community still lacks a standard protocol for determining $h$, we perform a sensitivity analysis to demonstrate that the resulting SPDs for radiocarbon and tree-ring dates stabilize at a cut width of 100 or so. We calculated SPDs at 25-year intervals from 0 (only duplicate dates are binned) to 150 years (effectively binning by site).

As can be seen in Figure 1, binning in general has little impact on the resulting SPDs, but cut widths above $h=100$ years are virtually indistinguishable. The shape of the radiocarbon SPD (panel A) stabilizes above $h=0$ and its values stabilize by $h=100$. The tree-ring SPD arguably stabilizes in both shape and value above $h=75$ or so, but in the analysis we use $h=100$ to keep our methods consistent between the radiocarbon and tree-ring SPDs.


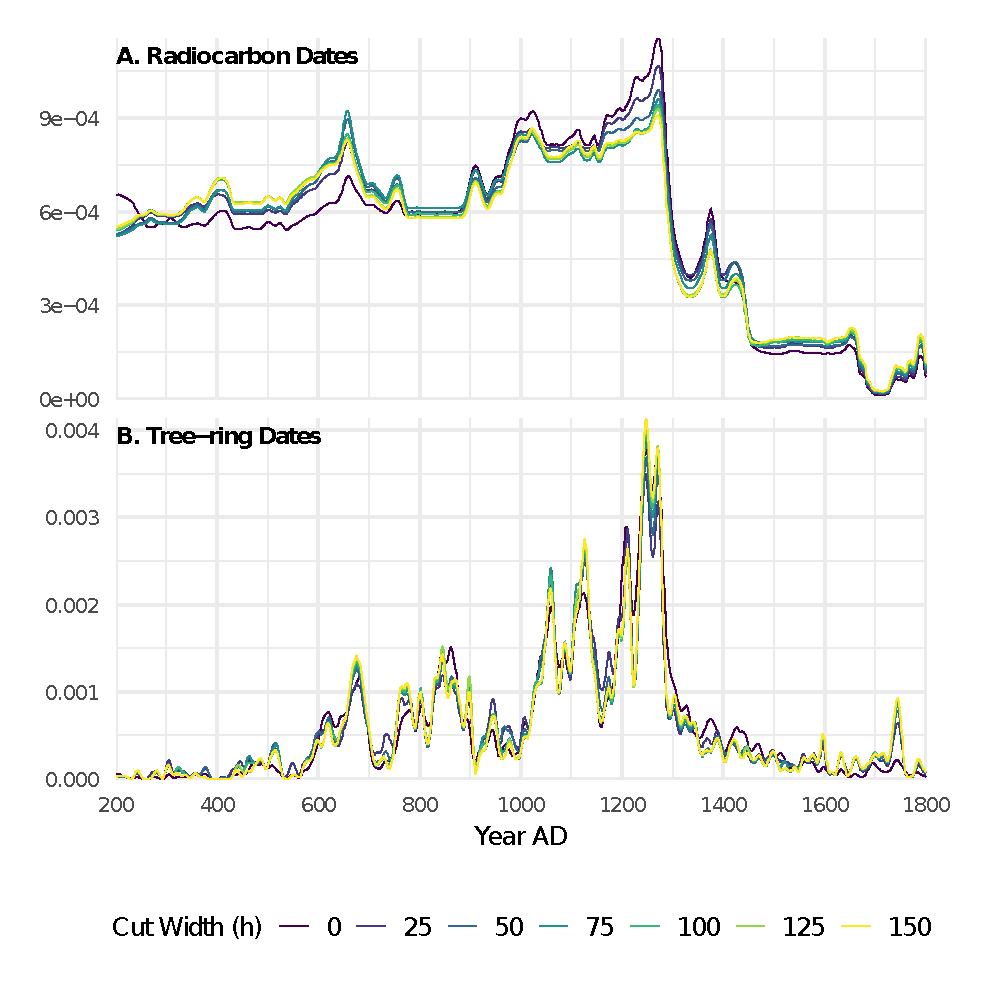


*Figure 1: Radiocarbon (A) and tree-ring (B) density distributions in the UUSW, given varying cut widths for binning dates within sites. The density distributions for the UUSW are normalized. Years are given in AD.*

# Coding for Dendrochronological Date Determinations

Dendrochronological date determinations customarily include several metadata tags that determine the quality of the date and the evidence supporting cutting versus non-cutting [2]. These codes include:

- - B: bark present.
  - G: beetle galleries are present on the surface of the specimen.
  - L: a characteristic surface patination and smoothness, which develops on beams stripped of bark, is present.
  - c: the outermost ring is continuous around the full circumference of the specimen; symbol is used only if a full section is present.
  - r: less than a full section is present, but the outermost ring is continuous around available circumference.
  - v: subjective judgement that, although there is no direct evidence of the true outside on the specimen, the date is within a very few years of being a cutting date
  - vv: there is no way of estimating how far the last ring is from the true outside.
  - +: one or more rings may be missing near the end of the ring series whose presence or absence cannot be determined because the specimen does not extend far enough back to provide an adequate check.
  - ++: a ring count is necessary due to the fact that beyond a certain point the specimen could not be dated.

# References

1. Bevan A, Crema ER. 2020 *rcarbon: Methods for calibrating and analysing radiocarbon dates*. See [https://CRAN.R-project.org/package=rcarbon](https://cran.r-project.org/package=rcarbon).
2. Laboratory of Tree Ring Research. Undated. *Archaeological Research: Explanation of Symbols*. University of Arizona. <https://www.ltrr.arizona.edu/archaeology/explsymbols.pdf>. Accessed 19 October 2020.
